# Prevention of Drinking and Driving

**Published:** 1996

**Authors:** Ralph Hingson

**Affiliations:** Ralph Hingson, Sc.D., is a professor and chair of the Social and Behavioral Sciences Department, Boston University School of Public Health, Boston, Massachusetts

**Keywords:** drinking and driving, deterrence of AODU (alcohol and other drug use), community based prevention, community based intervention, legislation, DWI laws, minimum drinking age laws, traffic accident, AODR (alcohol and other drug related) accident mortality, impaired driver, BAC, mandatory treatment, license revocation, imprisonment, probation, law enforcement, recidivism

## Abstract

Since the early 1980’s, legislative initiatives, such as the minimum legal drinking age of 21, administrative license revocation, and lower legal blood alcohol concentration limits for youth and adults, have significantly decreased alcohol-related traffic fatalities. General deterrence legislation is aimed at dissuading the general public from driving after drinking, whereas specific deterrence laws seek to prevent people who have been convicted for driving under the influence from repeating their offense. Education, enforcement, and comprehensive community programs, combined with legislation, can substantially reduce alcohol-related traffic deaths.

Traffic crashes are the leading cause of death in the United States for persons ages 1 to 34 (Insurance Institute for Highway Safety 1995). In 1995, 41 percent of fatal traffic crashes involved a driver or pedestrian who had been drinking (National Highway Traffic Safety Administration [NHTSA] 1996). This article reviews the nature and extent of the drinking-driving problem in the United States, discusses major legal and community initiatives in the past 15 years to reduce the problem, and examines other potential interventions for further reducing injuries and fatalities from alcohol-impaired driving.

## Alcohol and Impairment

Even at blood alcohol concentrations (BAC’s) as low as 0.02 percent, alcohol affects driver performance by reducing reaction time and slowing the decisionmaking process. Epidemiological research comparing BAC’s of drivers in single-vehicle fatal crashes with those of drivers stopped at random in nationwide surveys indicates that each 0.02-percent increase in BAC nearly doubles a driver’s risk of being in a fatal crash (Zador 1991). The risk increases more rapidly with each drink for drivers under age 21, who have less experience in driving and who, as a group, more often take risks in traffic, such as speeding or failing to wear seatbelts. For all groups of drivers, fatal crash involvement per miles driven increases ninefold at BAC’s of 0.05 to 0.09 percent (Zador 1991).

## Problems Posed by Alcohol-Impaired Driving

In 1995 there were 17,274 alcohol-related traffic fatalities and approximately 300,000 persons injured in alcohol-related crashes (NHTSA 1996*b*). Young people, people previously convicted for driving under the influence (DUI), and males in general are disproportionately involved in alcohol-related traffic deaths. Approximately 3 in 5 Americans will be involved in an alcohol-related crash at some point in their lives (NHTSA and National Institute on Alcohol Abuse and Alcoholism [NIAAA] 1996). In addition, alcohol-impaired driving often has an impact on innocent victims. In 1995, 39 percent of people killed in crashes involving drivers who had been drinking were persons other than the drinking driver.

Alcohol-related traffic crashes cost society $45 billion annually in hospital costs, rehabilitation expenses, and lost productivity (NHTSA 1995*a*). In 1995 more than 1.4 million people were arrested for driving while intoxicated, nearly 10 percent of all arrests made that year.

## Recent Trends in Alcohol-Related Traffic Deaths

The United States, like several other countries, has experienced marked declines in recent years in the number of fatal crashes involving alcohol. In 1982, when NHTSA began estimating the proportion of fatal crashes nationwide that involved alcohol, 25,165 fatal crashes (57.2 percent of all fatal crashes) involved a driver or pedestrian who had been drinking. In 1995 alcohol was involved in 17,274 crash fatalities, or 41.3 percent of all crash fatalities. Between 1982 and 1995, the proportion of crash fatalities involving alcohol fell by 28 percent, and the number of crash fatalities involving alcohol dropped by 31 percent (NHTSA 1996*a*).

Many improvements in traffic safety have occurred since 1982, such as the adoption of laws requiring the use of child restraints in all States and the enactment of legislation mandating the use of seatbelts in 49 States. However, the decline in alcohol-related traffic deaths was independent of these laws: Traffic deaths that did not involve alcohol increased 28 percent from 18,780 in 1982 to 24,524 in 1995.

The greatest declines in alcohol-related traffic deaths were among youth under 21. Among people ages 15 to 20, alcohol-related traffic deaths declined by 59 percent between 1982 and 1995, from 5,380 to 2,201. In this age group, the proportion of fatalities involving alcohol declined from 63 percent in 1982 to 36 percent in 1995, a 43-percent decline. Despite the long-term decline since 1982 in alcohol-related traffic deaths, a 4-percent increase in such deaths (from 16,589 to 17,274) occurred between 1994 and 1995, the first increase in 10 years. The increase occurred among persons age 21 and over.

## Grass Roots Activism

A key social change behind efforts to reduce drunk driving was the establishment in the early 1980’s of grass roots organizations such as Mothers Against Drunk Driving (MADD), Students Against Drunk Driving (SADD), and Remove Intoxicated Drivers (RID). Along with its support for victims affected by drunk drivers and its public education activities, MADD monitors research findings and, through legislative compendia, workshops, and national report cards rating the States, presents findings from this research and other testimony to State legislators. According to NHTSA (1996*a*), since 1982 more than 2,000 State laws have been passed in an effort to reduce alcohol-impaired driving. MADD has been an important force behind many of those laws.

## Effects of Laws To Reduce Alcohol-Impaired Driving

### Methodological Problems

Several factors make it difficult for researchers to isolate the effects of specific laws in reducing drunk driving. First, several laws are often passed in a given State within a relatively short time period, making it difficult to separate fully the effects of each law. Second, publicity about the drunk-driving problem is aired across State borders, diminishing any differences that may be found between States with different legislation.

Third, because most States do not determine BAC’s of all drivers involved in fatal crashes, researchers often rely on surrogate (i.e., proxy) measures of alcohol involvement in fatal crashes, such as single-vehicle nighttime (SVN) fatal crashes. SVN fatal crashes are three times more likely than other fatal crashes to involve alcohol. However, this proxy measure can be conservative: SVN fatal crashes account for less than one-half of all fatal traffic crashes involving intoxicated drivers. Consequently, the use of surrogate measures introduces some imprecision in evaluating the effects of legislation, particularly in short-term studies involving small jurisdictions. Variability in outcome measures from study to study makes cross-study comparisons difficult.

Fourth, people who drive after heavy drinking (defined as five or more drinks at one sitting) are much more likely to engage in other risky driving behaviors, such as speeding, running red lights, making illegal turns, driving after other drug use, and failing to wear seatbelts. Therefore, studies should control for shifts in legislation, targeting these behaviors (i.e., increased enforcement of traffic laws) as well as measuring overall changes in these behaviors.

Despite these methodological concerns, a number of conclusions can be reached concerning the effects of various legislative interventions in reducing alcohol-impaired driving.

## Major Legislative Changes To Reduce Alcohol-Impaired Driving

Legal efforts to reduce alcohol-impaired driving have emphasized deterrence. Deterrence laws seek to prevent alcohol-involved driving—through swift, certain, and severe penalties if warranted. Deterrence laws fall into two categories: (1) general deterrence laws, which aim to prevent the general public from ever driving after drinking, and (2) specific deterrence laws, which seek to prevent convicted DUI offenders from repeating their offense. Although convicted DUI offenders are at greater risk than other drivers for subsequent rearrest and crash involvement, most drivers in fatal crashes involving alcohol have never been previously convicted. In fact, two-thirds of persons arrested for DUI have never been arrested before (NHTSA 1995*a*,*b*). This statistic underscores the important need for laws and programs aimed at both general and specific deterrence.

### General Deterrence Laws

#### MLDA of 21

In all States it is currently illegal to sell alcohol to persons under age 21. Numerous research studies indicate that raising the minimum legal drinking age (MLDA) to 21 reduces alcohol-related fatal crash involvement among youth. States adopting MLDA’s of 21 in the early 1980’s experienced a 10- to 15-percent decline in alcohol-related traffic deaths among drivers in the targeted ages, compared with States that did not adopt such laws. NHTSA (1995*a*) estimated that MLDA’s of 21 have prevented more than 14,800 traffic deaths since 1976, approximately 700 to 1,000 deaths annually for the past decade. MLDA laws not only have reduced drinking among persons under age 21, they also have lowered drinking among people ages 21 to 25 who grew up in States with MLDA’s of 21 relative to those who grew up in other States (O’Malley and Wagenaar 1991).

Other nations that do not have an MLDA of 21 have experienced declines in alcohol-related fatalities among drivers under 21 (National Research Council of the Transportation Research Board 1994), as have many States that did not initially adopt an MLDA of 21. However, the evidence is clear and rather consistent that U.S. States which raised the legal drinking age experienced greater declines in fatal crashes likely to involve alcohol among drivers under 21 than did States that initially retained lower drinking ages (General Accounting Office 1987).

#### Criminal Per Se Laws

Every State except Massachusetts and South Carolina has adopted laws that make it a criminal offense per se to drive with a BAC above the State’s legal limit, which is generally either 0.10 or 0.08 percent. The per se provision means that prosecutors do not have to introduce evidence other than BAC to demonstrate impairment, making convictions easier to obtain.

#### Administrative License Suspension

This law allows a police officer or other official to immediately confiscate the license of a driver whose BAC exceeds the legal limit. License actions thus occur closer to the time of infraction and, by bypassing the court, are more swift and certain. Although these laws have faced some challenges for allegedly imposing double jeopardy on a driver who subsequently is convicted of DUI and receives additional penalties, no State supreme court has upheld such a challenge. In 1988 the Insurance Institute for Highway Safety conducted a nationwide comparison of administrative revocation laws, criminal per se laws, mandatory jail laws, and community service laws passed between 1978 and 1985 (Zador et al. 1989). Administrative license revocation laws were accompanied by a 5-percent decline in fatal crashes, compared with a 2-percent decline for other types of laws, such as criminal per se laws.

#### Zero-Tolerance Laws

Thirty-seven States and the District of Columbia now have adopted zero-tolerance legislation, laws that make it illegal for drivers under 21 to drive after drinking any alcohol. Laws setting legal BAC limits of 0.00 to 0.02 percent are considered zero-tolerance laws. A recent study compared the first 12 States that lowered legal BAC’s for drivers under 21 with 12 nearby States that did not. States adopting zero-tolerance laws experienced a 20-percent greater decline in the proportion of SVN fatal crashes among 15- to 20-year-old drivers. States lowering BAC limits to 0.04 or 0.06 did not experience significant declines relative to comparison States in the proportion of SVN fatal crashes among this age group (Hingson et al. 1994). The study projected that if the remaining States adopted zero-tolerance laws and experienced comparable declines, 375 to 400 fewer fatal cashes would occur each year. Zero-tolerance laws may be effective because they convey a clear message to youth: Drivers under 21 may not drive after any alcohol consumption whatsoever.

One problem encountered in implementing these laws has been the difficulty in achieving broad awareness of the law. Studies in California and Massachusetts found that 45 to 50 percent of young drivers were unaware of the law. Blomberg (1992), in a quasi-experimental study of an awareness campaign involving public service announcements about Maryland’s zero-tolerance law, found a one-third greater decline in alcohol-involved crashes among drivers receiving intensive education than among drivers who received no intensive education.

#### The 0.08-Percent Per Se Laws for Drivers Over Age 21

Thirteen States have adopted criminal per se laws lowering the legal BAC from 0.10 to 0.08 percent. Massachusetts has set the BAC for its administrative license revocation law at 0.08 percent.

Johnson and Waitz (1995) monitored six different measures of driver alcohol involvement in the first five States to adopt 0.08-percent per se laws and identified several statistically significant pre- to post-law decreases. However, comparison areas with different per se limits were not included; the study therefore could not determine whether post-law declines were independent of general regional trends.

A subsequent analysis (Hingson et al. 1996*a*) paired the first five States to adopt a 0.08-percent legal BAC limit with five nearby States that retained the 0.10-percent legal limit. As a group, States that adopted the 0.08-percent limit experienced a statistically significant 16-percent post-law decline in the proportion of fatal crashes involving fatally injured drivers at 0.08-percent BAC and higher. They also experienced a statistically significant 18-percent post-law decline in the proportion of fatal crashes involving fatally injured drivers at 0.15-percent BAC and higher. Four of the five 0.08-percent States experienced greater post-law declines than did comparison States. In the three largest of the five 0.08-percent States, the declines were significantly greater than in their respective comparison States. Those three pairs of States accounted for more than 90 percent of the fatal crashes in the study. During the pre- and post-law period, more than 80 percent of fatally injured drivers were given blood alcohol tests in 0.08-percent States.

Compared with 0.10-percent States, the 0.08-percent States may have been more concerned about alcohol-impaired driving and more responsive to legislative initiatives to reduce the problem. All five 0.08-percent States had administrative license revocation laws during the study period, three of which were implemented within 1 year of the 0.08-percent law. Administrative license revocation laws have been associated with a 5-percent decline in fatal crashes and as much as a 9-percent decline in alcohol-related fatal crashes (Klein 1989). This potential effect restricted the study’s ability to separate the effect of 0.08-percent laws from that of administrative license revocation laws. Only in Maine was administrative license revocation in place throughout the pre-law analysis period prior to adoption of the reduced BAC limit. Thus, Maine was the only State where it was possible to identify an independent effect of the 0.08-percent law.

The results of this study suggest that 0.08-percent laws combined with administrative license revocation can reduce the proportion both of fatal crashes involving drivers and of fatally injured drivers at 0.08-percent BAC or higher and at 0.15-percent BAC or higher. The study projected that if all States adopted 0.08-percent laws—with results similar to those of the first five States to adopt such laws—at least 500 to 600 fewer deaths would occur on the Nation’s roadways each year.

In 1994 Massachusetts simultaneously introduced a 0.08-percent BAC and administrative license revocation laws. Statewide randomized telephone surveys in 1993 (i.e., before the 0.08-percent BAC law was implemented) and in 1996 (i.e., after the law was implemented) revealed clear shifts in public perceptions of how much drivers could drink and still drive safely and legally. The proportion of respondents who believed they could consume four or more drinks and drive safely declined from 24 to 15 percent, and the proportion who felt they could drive legally after more than four drinks dropped from 18 to 9 percent. At the same time, the proportion of respondents who believed drunk drivers would have their license suspended before a trial rose from 47 to 71 percent. In addition, the proportion of drivers who reported driving in the past month after consuming four or more drinks declined from 9 to 4 percent (Massachusetts Governor’s Highway Safety Bureau 1996).

### Specific Deterrence Laws

Persons convicted of alcohol-impaired driving are more likely than other drivers to be subsequently arrested for driving while intoxicated and to be involved in alcohol-related crashes (NHTSA and NIAAA 1996). Specific deterrence laws seek to reduce this recidivism through license actions, treatment or rehabilitation, jail sentences, dedicated detention, probation, actions against vehicles and vehicle tags, lower legal BAC’s for offenders, or some combination of these measures.

#### License Actions

Mandatory license suspensions are more effective than discretionary suspension in reducing total crashes and violations. Their effectiveness is attributed to the fact that the laws generate a perceived certainty of punishment and reduce the influence of judicial discretion. Evidence also indicates that diversion to treatment with either a restricted or a limited license leads to higher accident and violation rates than full license suspension. Several studies also report that full license suspension reduces DUI recidivism. Much of this benefit may result from reduced driving exposure.

NHTSA and NIAAA (1996) prepared *A Guide to Sentencing DUI Offenders* and reported the following:

- Suspension periods between 12 and 18 months appear to be optimal for reducing DUI recidivism.
- Suspension periods of less than 3 months seem to be ineffective.
- Although more than 50 percent of persons with suspended licenses continue to drive, they seem to drive less frequently and more cautiously in order to avoid arrest.

#### Treatment or Rehabilitation

Wells-Parker and colleagues (1995) recently completed a meta-analysis of treatment efficacy for DUI offenders. Compared with standard sanctions (i.e., jail or fines) or no treatment, rehabilitation generated a 7- to 9-percent reduction in the incidence of alcohol-related driving recidivism and crashes when averaged across all types of offenders and rehabilitation. Alcohol-related crashes and violations constitute only a minority of total crashes and violations. Actions to restrict license use (e.g., daytime-only driving permits) combined with some kind of remedial treatment have been found to be more effective in preventing alcohol-related traffic incidents than full suspension.

The analysis by Wells-Parker and colleagues (1995) also indicated that treatments combining punishment strategies, education, and therapy with followup monitoring and aftercare were more effective for first-time as well as repeat offenders than any single approach. For example, combining treatment with licensing action was more effective than either tactic alone. According to this analysis, treatment alone never substitutes for sanctions or remedies, and remedies and sanctions do not substitute for treatment. Finally, weekend intervention programs designed to evaluate alcohol and other drug abuse and create an individualized treatment plan for offenders have been found to produce lower recidivism rates than jail, suspended sentences, or fines.

#### Jail Sentences

Although incarceration incapacitates drivers during the period of confinement, minimal evidence exists on the postincarceration effect of jail. Nichols and Ross (reviewed in NHTSA and NIAAA 1996) examined the specific deterrent effects of jail sentences for first-time and repeat DUI offenders in a review of more than 80 studies of legal deterrence. Eight studies reported no reductions in DUI recidivism as a result of jail sentences, and only one recent study provided reasonably convincing evidence of a 3-year reduction for first-time repeat offenders who received mandatory 2-day jail sentences in Tennessee. In one study, long periods of incarceration were actually associated with higher recidivism (Mann et al. 1991). Although jail sentences may have some short-term general deterrent effects, as well as deterrent effects for first-time offenders, mandatory jail sentences tend to affect court operations and the correctional process negatively by increasing the demand for jury trials and plea bargains and by crowding jails (NHTSA and NIAAA 1996).

#### Dedicated Detention

Detention facilities specifically for DUI offenders can offer both incapacitation and supervised rehabilitation services. One program of this type, in Prince Georges County, Maryland, has been found to reduce recidivism among both first-time and repeat offenders (Harding et al. 1989).

#### Probation

The 1996 NHTSA–NIAAA review of sentencing options found that probation may slightly reduce recidivism among drivers at low risk for being repeat offenders, but probation alone does not measurably reduce recidivism among those at high risk. Although the effects of intensive probation and home detention have not been evaluated, a 7-year study of electronic monitoring found that recidivism rates were less than 3 percent for a group of DUI offenders who were electronically monitored during 203 months of their combined probation. However, recidivism increased when the monitoring ended.

#### Actions Against Vehicles and Tags

Although license actions have been shown to reduce recidivism, many people with suspended licenses continue to drive.

Unlicensed drivers can be apprehended only when police have probable cause to stop their vehicle. Washington and Oregon have enacted legislation that allows police to seize the vehicle registration of drivers caught driving after suspension, leaving the motorist with a temporary, 60-day registration. A sticker on the vehicle gives the police probable cause to stop the vehicle to ask for proof of license. Researchers have reported evidence of the law’s effectiveness in Oregon but not in Washington.

Rodgers (1994) measured the effectiveness of the 1988 license-plate impoundment law for one-third of the DUI offenders in Minnesota. During the 29 months that the courts administered the system, only 6 percent of 7,698 eligible third-time offenders had their license plates impounded. During the 21 months in which the law was managed through the Department of Public Safety, 68 percent of the 4,593 third-time DUI offenders had vehicle plates impounded. The law had little deterrent effect while the courts administered the system. In contrast, when the program was managed administratively, offenders who lost their plates had a lower rate of recidivism than those who did not.

Another approach—ignition interlocks to prevent vehicle operation when a driver’s breath alcohol exceeds a designated limit—has been found to reduce recidivism, but recidivism may rise after the device is removed.

The NHTSA–NIAAA (1996) sentencing guide identified several other sentencing approaches that have not been systematically evaluated, including financial sanctions, publication of offenders’ names in newspapers, attendance at victim-impact panels, victim-restitution programs, and court-ordered visits to emergency rooms.

#### Lower Legal Blood Alcohol Limits for Convicted DUI Offenders

Despite the fact that persons convicted of DUI are more likely than other drivers to be subsequently arrested for DUI or to be involved in crashes, almost all States allow the same legal BAC for these drivers as for drivers who have never been convicted of DUI. One exception is Maine. In 1988 the State set the legal limit at 0.04 percent for drivers previously convicted for DUI, lower than the 0.08 percent limit for other drivers. In the 3 years following enactment of the law, nighttime fatal crashes involving drivers with prior convictions declined by 38 percent, whereas such crashes increased by 50 percent in neighboring New Hampshire and Vermont (Hingson 1995).

#### Enforcement of Impaired Driving Laws

The extent to which drunk-driving laws are enforced can influence their impact on impaired driving. Drunk-driving arrests increased dramatically between 1978 and 1983, from 1.3 to 1.9 million, but arrests have dropped each year since then, to 1.4 million in 1994. Estimates indicate that only 1 arrest is made for every 300 to 1,000 drunk-driving trips (Voas and Lacey 1989). Respondents in a 1995 national survey of 4,000 randomly selected drivers believed that people who drink and drive are more likely to be in an accident than to be stopped by the police. Only 23 percent of the respondents thought it very likely that people who drive after drinking will be stopped by the police, down from 26 percent in 1993 (NHTSA 1996*a*).

The most dramatic example of the potential deterrent impact of police enforcement of drunk-driving laws occurred in Australia in New South Wales and Victoria, where random breath testing was introduced on a massive scale. In a given year, as many as 1 driver in 3 was stopped by the police. There was an immediate 37-percent drop in alcohol-related fatal crashes, compared with the previous 3 years, and a sustained 24-percent decrease over the next 5 years (NHTSA and NIAAA 1996).

In the United States, police do not have the authority to administer breath tests to individual drivers who have been stopped unless there is probable cause to believe that the driver is under the influence of alcohol. Instead, police must use surveillance by police patrols or sobriety checkpoints, often at pre-designated, high-risk areas and involving several patrol officers. Several evaluations of sobriety checkpoints have demonstrated their effectiveness. One recent California study found that checkpoints reduced alcohol-related crashes regardless of the way in which they were implemented (e.g., using from 3 to 5 officers versus using from 8 to 12 officers) or whether the officers remained at one location for an entire evening or moved to multiple locations.

Although checkpoints have considerable deterrence potential, they are limited in that many drunk drivers pass through roadblocks undetected. Research involving checkpoints where drivers not detained by police were subsequently tested for alcohol indicates that about one-half of the drivers with BAC’s above the legal limit are not detained (Ferguson et al. 1995).

Passive alcohol sensors increase detection of drunk drivers in sobriety checkpoints. Passive sensors collect air from in front of a driver’s face and can detect the presence of alcohol in the driver’s breath. Use of these sensors is not believed to constitute a search under the fourth amendment and can be used by officers to establish probable cause that a driver has been drinking.

In one recent study, police detected 55 percent of drivers above the legal BAC limit when not using passive sensors, compared with 71 percent when using sensors (Ferguson et al. 1995). In addition, Jones and Lund (1986) found that when sensors are used, sober drivers are less likely to be erroneously suspected of alcohol use, and a lower percentage are asked to take field sobriety tests.

Perhaps the most extensive Statewide sobriety checkpoint program in the United States was implemented in Tennessee. From April 1994 through March 1995, more than 150,000 drivers were stopped at 900 checkpoints. The program was highly publicized on television. A quasi-experimental study revealed a 17-percent reduction in alcohol-related fatal crashes in Tennessee relative to five contiguous States during the same time period (Lacey et al. 1996).

Preliminary breath testers have been available to police for more than 20 years. They are generally used after the police officer has conducted a field sobriety test and are useful in establishing probable cause for intoxication. States with laws permitting the use of preliminary breath tests at the roadside have been found to reduce nighttime fatal crashes significantly, even after the results were controlled analytically for a dozen potential confounding variables such as unemployment, income, alcohol taxes, miles driven, and drinking.

#### Comprehensive Community Interventions

Enforcement is most likely to be effective in deterring alcohol-impaired driving if it is publicized, and it is most likely to be actively pursued by the police if they feel there is a strong demand for such action.

Citing its long-term success with other public health problems, the Institute of Medicine of the National Academy of Sciences has recommended comprehensive multistrategy community interventions to reduce alcohol-related health problems. For example, comprehensive community programs have achieved some success in reducing cardiovascular mortality and risks such as fat intake, blood pressure, smoking, and cholesterol level. Some recent community interventions have achieved minimal reductions in cardiovascular risks and prevalence of smoking; however, one study found significant declines in unintentional childhood injury.

The Saving Lives Program was implemented in March 1988 (Hingson et al. 1996*b*). In each of six Massachusetts cities (combined population of 318,000), a full-time coordinator from the mayor’s or city manager’s office organized a task force of concerned private citizens and organizations and officials representing various city departments (e.g., school, health, police, and recreation). Each community received approximately $1 per inhabitant annually in program funds. Active task-force membership ranged from 20 to more than 100 persons, and an average of 50 organizations participated in each city. The communities not only attempted to reduce alcohol-impaired driving but also targeted other risky driving behaviors that alcohol-impaired drivers are more likely to engage in, such as speeding, red light violations, failure to yield to pedestrians in crosswalks, and failure to wear seatbelts.

Fatal crashes in program cities declined from 178 in the 5 years before the program to 120 during the 5 program years, a 25-percent decrease relative to the rest of Massachusetts. Likewise, fatal crashes involving alcohol declined by 42 percent relative to the rest of the State, from 69 in the 5 years preceding the program to 36 during the 5 years of the program. The number of fatally injured drivers with positive BAC’s showed a decline of 47 percent in program cities, from 49 to 24. Visible injuries per 100 crashes declined by 5 percent, from 21.1 to 16.6. The proportion of vehicles observed speeding and the proportion of teenagers who reported driving after drinking were cut in half. The results clearly indicate that interventions organized by multiple city departments and private citizens can reduce driving after drinking, related driving risks, and traffic deaths and injuries. A major question is whether these changes can be sustained without support from the initial grant sources.

In the Community Trials Program (Holder et al. in press), three experimental communities—one each in northern and southern California and one in South Carolina—were paired with comparison communities. The program incorporated community mobilization, media advocacy, training of alcoholic beverage servers, development of written serving policies by bars and restaurants, local zoning to reduce alcohol-outlet density, local enforcement of underage alcohol sales, alcohol-outlet clerk training in asking for age identification, police officer training, additional officer enforcement hours, use of passive alcohol sensors, and monthly sobriety checkpoints.

In program communities, relative to comparison communities, changes included statistically significant program-related increases in media coverage of alcohol issues in local newspapers and on local television, a significant reduction in alcohol sales purchases to minors (i.e., sales were cut in half), and increased adoption of responsible server policies. Across all three program communities, a significant 10-percent postprogram reduction occurred in the numbers of SVN crashes per 100,000 population, with the greatest effects in the two California communities.

The results from these two studies reinforce findings from an earlier community study in which bartenders and counter clerks were trained to demonstrate the use of calculators to identify customer BAC levels based on customer weight and the number of drinks consumed. Television spots reinforced the messages. Roadside surveys conducted in both the intervention and the comparison communities before the program was implemented and 6 months after it was implemented revealed no differences between communities before the program was initiated. At the 6-month survey, 5.8 percent of nighttime drivers in the program communities had BAC’s of 0.05 percent or higher, compared with 11.1 percent in the comparison communities. This study demonstrates the potential for community interventions to change social norms about unacceptable drinking behavior before driving.

## Potential Areas of Research

Despite the progress made in reducing alcohol-related crashes since the early 1980’s, the increase in such crashes in 1995 reminds us that important research questions persist.

### Enforcement Decline: Cause and Effects

Arrests for drunk driving have declined 26 percent since 1983. The reasons for those declines are not clear. Whether they reflect changes in attitudes among police command staff and patrol officers searching for DUI offenders or in the exercise of discretion in arresting offenders warrants exploration. The impact of reduced arrest rates on drivers’ perceptions of the likelihood that alcohol-impaired drivers will be arrested also deserves scrutiny.

### Enforcement of Related Traffic Laws

People who drive after drinking alcohol are more likely than other drivers to speed, run red lights, fail to yield to pedestrians, and fail to wear seatbelts. All of these behaviors heighten the risk of crashing or of being injured in a crash. The community program reported by Hingson and colleagues (1996*b*) in Massachusetts targeted not only alcohol-impaired driving but also these related driving behaviors. Whether this strategy can succeed in other States warrants exploration. Massachusetts did not have a seatbelt law during that study period. Combining DUI enforcement with seatbelt checkpoints should be explored, particularly in States with primary seatbelt law enforcement that permit police to stop a vehicle because occupants are unbelted. Similarly, combining DUI and speed enforcement warrants study, because a high proportion of fatal crashes involving alcohol also involve excessive speed.

#### Pedestrians and Alcohol

In 1995, 5,585 pedestrians died in alcohol-related crashes. Twenty percent of the drivers and 37 percent of the pedestrians had been drinking. Most attention on alcohol-involved traffic crashes has focused on drivers. Jones et al. (1992) found that adopting the MLDA of 21 reduced the number of both teenage driver and pedestrian deaths. Whether policy initiatives, such as lower legal BAC limits, alcohol taxes, zoning, or altering hours of sale, can influence pedestrian deaths deserves study.

#### General Transportation Changes

Ross (1992) has indicated that most legal and community approaches to date have emphasized legal deterrence of alcohol-impaired driving. Less political discussion has focused on social causes of impaired driving. Ross insists that impaired driving can also be reduced by policies that decrease the overall use of cars as well as of alcohol. In addition, other strategies should be explored, including taxes and regulations on vehicles, gasoline, and alcohol sales; improved public transportation; and restrictions on driver age. Ross has also urged an examination of efforts to attenuate links between impairment, error, and crashes and between crashes, injuries, and deaths. Examples include improved highway and vehicle engineering and improved emergency medical services.

## Conclusion

Since the early 1980’s, legislative initiatives, such as the MLDA of 21, administrative license revocation, and lower legal BAC limits for youth and adults, have been independently associated with significant declines in alcohol-related traffic deaths. Active education and enforcement programs can enhance the beneficial effects of these laws. Furthermore, comprehensive community interventions that integrate the efforts of several city Government departments with those of concerned private citizens and organizations can substantially reduce alcohol-related traffic deaths, particularly if the programs also devote attention to other risky traffic behaviors disproportionately found among drinking drivers (e.g., speeding, running red lights, and failure to wear seatbelts). Nonetheless, although increases in the implementation of MLDA laws, traffic laws, and programs have helped cut alcohol-related traffic deaths by 31 percent nationwide, the bulk of the problem persists: Alcohol-related traffic deaths increased 4 percent in 1995, the first increase in a decade. Research is needed to explore ways to increase police enforcement of existing drunk-driving laws, to foster the passage of legislation known to reduce traffic injury and death, and to explore new ways to reduce alcohol-related traffic deaths, not only among drivers and passengers, but also among pedestrians. Furthermore, new ideas and new approaches will be needed if we are to make dramatic additional advances addressing this major public health problem.
